# Bioactive Compounds of *Ganoderma boninense* Inhibited Methicillin-Resistant *Staphylococcus aureus* Growth by Affecting Their Cell Membrane Permeability and Integrity

**DOI:** 10.3390/molecules27030838

**Published:** 2022-01-27

**Authors:** Yow-San Chan, Khim-Phin Chong

**Affiliations:** Faculty of Science and Natural Resources, Universiti Malaysia Sabah, Jalan UMS, Kota Kinabalu 88400, Sabah, Malaysia; mz1621052t@ums.edu.my

**Keywords:** *G. boninense*, solid-phase extraction (SPE), antibacterial properties, LCMS, metabolites profile, mode of action

## Abstract

Some species of *Ganoderma*, such as *G. lucidum*, are well-known as traditional Chinese medicine (TCM), and their pharmacological value was scientifically proven in modern days. However, *G. boninense* is recognized as an oil palm pathogen, and its biological activity is scarcely reported. Hence, this study aimed to investigate the antibacterial properties of *G. boninense* fruiting bodies, which formed by condensed mycelial, produced numerous and complex profiles of natural compounds. Extract was cleaned up with normal-phase SPE and its metabolites were analyzed using liquid chromatography–mass spectrometry (LCMS). From the disc diffusion and broth microdilution assays, strong susceptibility was observed in methicillin-resistant *Staphylococcus aureus* (MRSA) in elute fraction with zone inhibition of 41.08 ± 0.04 mm and MIC value of 0.078 mg mL^−1^. A total of 23 peaks were detected using MS, which were putatively identified based on their mass-to-charge ratio (*m/z*), and eight compounds, which include aristolochic acid, aminoimidazole ribotide, lysine sulfonamide 11v, carbocyclic puromycin, fenbendazole, acetylcaranine, tigecycline, and tamoxifen, were reported in earlier literature for their antimicrobial activity. Morphological observation via scanning electron microscope (SEM), cell membrane permeability, and integrity assessment suggest *G. boninense* extract induces irreversible damage to the cell membrane of MRSA, thus causing cellular lysis and death.

## 1. Introduction

The fruiting body of the *Ganoderma* genus practically is not acceptable as an edible mushroom due to its woody texture and bitter taste. However, people, especially Asians, are fascinated with the medicinal values of these woody mushrooms and have been using them traditionally for remedy or treatment of various ailments [1]. In ancient times, the wild fruiting body of *Ganoderma* was prepared as a tonic, tincture in alcohol, or decoction in water for strengthening body resistance, improving eyesight and vital essence and also nourishing spleen and liver [2]. Over centuries, the *Ganoderma* mushroom has been historically accredited as “Mushroom of Immortality”, and it is believed it can enhance health, well-being, and longevity [3]. In the present day, the medicinal treasure chest of the *Ganoderma* genus in various species was deeply discovered and reported with the presence of numerous bioactivities [4,5,6]. For instance, the bioactive β-1-3-glucan polysaccharides isolated from the fruiting body of *G. lucidum* exhibited a broad range of bioactivities including antitumor, anticancer, anti-inflammatory, and immune-stimulating effects while various metabolites of triterpenes such as ganoderic acids and lucidenic acids demonstrated antibacterial, antifungal, antiandrogenic, anti-human immunodeficiency virus (HIV), antiplatelet aggregation effects, and others [7]. Recently, due to the acceptance of *Ganoderma*’s products as dietary supplements and herbal medicines by United States Pharmacopoeia [8], the cultivation of *Ganoderma* fruiting body has bloomed, and China was reported to export over 110,000 tons of fruiting bodies per year [9]. With the significant values of the fruiting body, the mycelium and spores of *Ganoderma* are also used as part of medicine [10,11]. Besides the well-studied *G. lucidum*, the fruiting bodies extract of *G. applanatum* and *G. australe* also demonstrated bacterial growth inhibition against *Escherichia* *coli*, *Pseudomonas syringae*, *Bacillus subtili, P. aeruginosa*, *B. cereus*, and *Staphylococcus aureus*. [12,13]. Bioactivities (antitumor, immune-modulatory, and hypocholesterolemic) have also been reported on *G. applanatum* [14]. However, unlike other *Ganoderma* spp., the studies on bioactivity of *G. boninense* are comparatively scarce. Some of the work on *G. boninense* bioactivity include the work of Ma et al. on antiplasmodial activity [15]; Ismail et al. reported antimicrobial activity of non-polar solvent extracts of *G. boninense* [16]; Chan and Chong reported on the antibacterial activity [17], phytochemical and antioxidant study [18] of crude extract of *G. boninense* fruiting body, while Abdullah et al. [19,20] reported the antibacterial activity of single compound isolated from *G. boninense* mycelia. This study revealed the importance of the further clean-up process to preconcentrate the targeted groups of metabolites for antibacterial activity from *G. boninense* fruiting body. To our best knowledge, this is the first investigation of the antibacterial mode of action of *G. boninense* fruiting body extract.

Current research on *G. boninense* is focusing on its pathogenesis study on palm trees [21,22] and controlling the spread of this fungus using various approaches [23,24]. To date, *G. boninense* remains the greatest threat to the oil palm plantation due to its enormous devastation towards palm trees by causing incurable basal stem rot (BSR) disease after invading and colonizing the tree. This soil-borne pathogen is a parasitic and saprophytic fungus that attacks the living tissue and decomposes the bole or lower stem of palm tree through colonization on the root surface, crossing the epidermal cells and spreading into cortical cells and vascular system [25].

On the other hand, the world is facing emergence in antimicrobial resistance (AMR), and this issue has resulted in difficulty in infection treatment and increased rate of mortality. According to the 2019 AMR Threats Report published by the Centers for Disease Control and Prevention (CDC), more than 2.8 million antibiotic-resistant infections occurred in the United States, which led to more than 35,000 deaths [26]. World Health Organization (WHO) estimated people with methicillin-resistant *Staphylococcus aureus* (MRSA) infection are 64% more likely to die [27]. Despite treating MRSA infection with different types of antibiotics, ample research on bioactive natural products was conducted to address this issue [28].

Crude extract of a natural product is a complex matrix due to the co-existing of many diverse metabolites in a mixture. This often extends to another issue where the compounds of interest are present in low concentrations [29]. Hence, the sample pre-concentration or clean-up is crucial to remove a large number of interfering constituents in the sample matrix while retaining the compounds of interest. Solid-phase extraction (SPE) is one of the most commonly used methods in sample clean-up or increasing the concentration of trace level analytes [30]. Technically, the concept of SPE is like liquid–liquid extraction (LLE), chemically separating the undesired constituents of the sample by partitioning of solutes between two immiscible liquid phases. SPE partitioning consists of a solid phase with the sorbent and the liquid phase where the sample matrix is filled in the cartridge column. Analytes that are retained on the solid phase are washed and eluted using a small amount of organic solvent, yielding concentrated and simplicated desired analytes [31].

In this study, the SPE method was performed on solvent extracts of *G. boninense* fruiting body, and the fractions were evaluated for their antibacterial properties. Fractions that exhibited good activity in bacteria growth inhibition were further separated using a liquid chromatography (LC) system, and the metabolites were identified using mass spectrometry (MS) based on the mass-to-charge (*m/z*) ratio. Furthermore, the effective minimum inhibition concentration (MIC) of the extract was used as treatment concentration to investigate its mode of action against selected Gram-positive bacteria through SEM morphological examination, measurement of intracellular leakage of relative electrical conductivity, and 260 nm absorbing materials.

## 2. Results and Discussion

### 2.1. Identification of the Metabolites from EF

**Figure 1:**
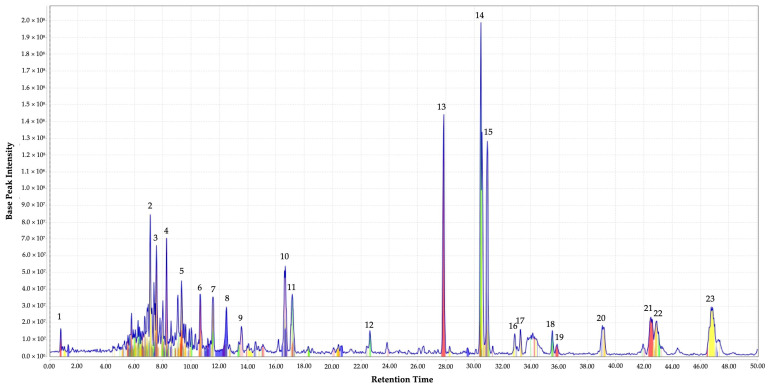
LC–Ion Trap–MS profile and extracted ion chromatogram (XIC) of ethyl acetate *G. boninense* SPE elute fraction (EF). Each colored peak represents detected individual peaks. Peak number is assigned based on elution order.

The metabolites profile of EF was illustrated by extracted ion chromatogram (XIC) in Figure 1. A total of 23 well-separated peaks, represented in colored peaks after chromatographic deconvolution, were detected at the retention time ranging from 0.7 to 47.0 min with different intensity and *m/z* values. All the detected peaks were tentatively assigned based on the *m/z* value (molecular weight) of the compounds obtained from LC–Ion Trap–MS analysis and mass search in Kyoto Encyclopedia of Genes and Genomes (KEGG) and PubChem database. The characteristics of detected peaks such as retention time, peak intensity, *m/z* value, putative identity, suggested molecular formula, and metabolite class are summarized in Table 1.

Based on the literature search with published works, eight of the metabolites from the current work were reported for their antimicrobial activity. Peak 6 was identified as aristolochic acid, which was known to be effective against Methicillin sensitive *S. aureus* [32]; peak 9 as aminoimidazole ribotide, which was reported to potentially play an important role in the development of antibacterial drugs [33]; peak 10 as lysine sulfonamide, which possesses antiviral, antifungal, and antibacterial properties [34,35]. Carbocyclic puromycin, which was reported as an aminonucleoside antibiotic [36], was also found as peak 12, while fenbendazole, which was documented as an anthelmintic drug but was repurposed for multidrug-resistant intention [37,38], was assigned to peak 14 in the current EF. Moreover, acetylcaranine (peak 16) was reported to be active against *Candida albicans*, *C. dubliniensis*, *C. glabrata*, and *Lodderomyces elongiosporus* [39]; tigecycline (peak 17) is a marketed antibiotic [40]. The last identified metabolites from EF with antimicrobial properties reported in the literature was tamoxifen (peak 19) which is effective against MRSA, *P. aeruginosa*, and *E. coli* [38,41]. Therefore, the combination of these metabolites in the EF may have contributed to the antibacterial activity recorded in the current work.

In addition to antimicrobial activity, some of the putatively identified metabolites of EF were also reported with other biological activities. For example, peptide pyroGlu-Gln-Gly-Ser-Asn inhibits DNA synthesis and proliferation of rat hepatoma cells [42]; idarubicinol and deglucohyrcanoside possess anticancer properties [43,44]; spiramine A and cilostazol were reported with antiplatelet activity [45,46]. Hence, we strongly believe that *G. boninense* extract has a wide potential in other biological activities.

Nonetheless, the untargeted metabolite identification based on *m/z* remained a major challenge due to the intensive chemical and physical diversities in metabolites. In our case, some synthetic compounds were putatively assigned due to the presence of compounds with extremely similar molecular weights, and mass-based identification cannot discriminate isomers that have the same elemental compounds but different structures [47]. However, the mass-based metabolic identification could be authenticated by tandem MS for metabolite identity verification.

### 2.2. Antibacterial Activity of G. boninense SPE Extract

#### 2.2.1. Bacteria Growth Inhibition

**Figure 2:**
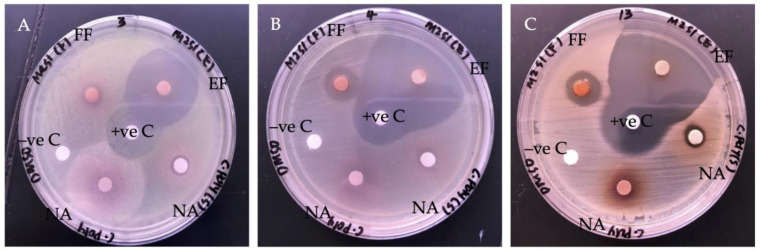
Images of the inhibitory test by disc diffusion with ethyl acetate *G. boninense* SPE extract: (**A**) *S. pyogenes*; (**B**) CoNS; (**C**) MRSA. All the agar plates included with Chloramphenicol (30 μg/disc) were used as positive control and 10% DMSO as negative control. Labels: flush fraction (FF); elute fraction (EF); not applicable (NA); positive control (+ve C); negative control (−ve C).

The antibacterial activity of SPE extract fractions was evaluated based on their ability in bacteria growth inhibition. Thus, a larger radius of the inhibition zone indicates more potent antibacterial activity. As two fractions of the SPE extract were evaluated, the fractions were labeled as flush fraction (FF) and elute fraction (EF). The antibacterial activity and the size of their inhibition zones against the tested bacteria are tabulated in Table 2 and illustrated in Figure 2. By comparing the size of the inhibition zones by both fractions, EF showed a greater diameter (mm) of inhibition zones. The inhibition zone of EF ranged from 30.75 ± 0.03 mm to 47.25 ± 0.07 mm. The highest growth inhibition was observed against Coagulase-negative *Staphylococci* (CoNS), followed by MRSA and *Streptococcus pyogenes* (*S. pyogenes*). Meanwhile, FF exhibited growth inhibition with a smaller diameter of inhibition zones ranging from 8.75 ± 0.04 mm to 20.42 ± 0.04 mm. Hence, the result showed that the EF might possess more potent antibacterial properties. According to the clinical breakpoints guidance provided by European Committee on Antimicrobial Susceptibility Testing (EUCAST), the inhibition zone of positive control, Chloramphenicol (30 μg/disc), was in the susceptible category against *Staphylococcus* spp. (≥18 mm), *Streptococcus* groups A, B, C, and G (≥19 mm) [48]. In addition, in our screening of antibacterial activity of EF, the extract fraction also showed growth inhibition against some Gram-negative bacteria, such as *Klebsiella pneumoniae* (35.33 ± 0.03 mm), *Serratia marcescens* (33.50 ± 0.05), *P. aeruginosa* (31.92 ± 0.01 mm), and *Proteus mirabilis* (24.25 ± 0.03 mm) [49]. Moderate inhibition was observed on the Gram-negative bacteria, which consist of an outer membrane that has been reported on resistance to antibacterial agents [50]. Moreover, the outer membrane also consists of outer membrane proteins, such as porins, to allow passive diffusion of small molecules at the same time regulate and protect the bacteria from toxicity via efflux mechanism [51]. However, the data were not published, as no further investigation was conducted.

The crude extract is a combination of complex mixtures with the presence of high complexity and difference in metabolites. Therefore, further purification is always needed for obtaining better biological activity [52]. The result in Table 2 reveals the normal phase SPE successfully retained and recovered the antibacterial compounds. Our study used normal phase SPE where metabolites in the extract with a similar chemical structure will be sorbed on the SPE cartridge, which is compacted with silica-based sorbent. The OH-group on the sorbent establishes hydrophilic interactions with compounds containing hydroxyl, carbonyls, amines, or alkenes functional group. Hence, polar analytes from the extract mixtures are mostly separated from the non-polar analytes, which are contained in a flush fraction [53]. Therefore, compounds responsible for the antibacterial activity in *G. boninense* SPE extract are mostly polar analytes. This fractionation technique provides high versatility and selectivity yet achieves high extraction efficiency of organic compounds compared to liquid–liquid extraction (LLE) [54]. SPE extraction technique has been utilized in the previous study for pre-concentration and clean-up of interest compounds such as peptides from lignocellulolytic enzyme of *G. lucidum* [55] and fractionation and detection of triterpenoid from *G. lucidum* [56].

#### 2.2.2. Determination of Minimum Inhibitory Concentration (MIC)

Since EF had demonstrated a greater bacteria growth inhibition compared to FF, the MIC of the fraction against bacterial strains was determined. The result in Table 3 showed that bacteria MRSA was inhibited at the lowest inhibitory concentration of 0.078 mg mL^−1^. Meanwhile, the rest of the test pathogens (CoNS and *S. pyogenes*) were inhibited at the concentration of 0.156 mg mL^−1^.

The result of MIC in this study was lower compared to the concentration of ethyl acetate crude extract of *G. boninense* used in our previous work [17]. This implied the selection of normal phase SPE was successfully preconcentrated the antibacterial compounds from mixtures of crude extract. In addition, the MIC of EF in this study was also lower in comparison to MIC of crude extracts of different species of *Ganoderma* such as *G. lucidum*, *G. australe,* and *G. pfeifferi* against some similar pathogens. Quereshi et al. [57] had recorded the lowest inhibitory concentration of *G. lucidum* acetone extract as 4.33 mg mL^−1^ against *K. pneumoniae*, while Yoon et al. [58] reported the lowest inhibitory concentration of aqueous extract of the same *Ganoderma* as 0.750 mg mL^−1^ against *Micrococcus luteus*. Lower MIC obtained in our current work could further suggest that SPE had successfully preconcentrated the antibacterial compounds in ethyl acetate crude extract. Comparatively, the EF also showed comparable MIC values with some of the purified antibacterial compounds from *G. australe* and *G. pfeifferi*, such as methyl australate and australic acid, which was reported with MIC of 0.250 mg mL^−1^ against *B. cereus* [13] and ganomycin A and B which was reported with MIC of 0.025 mg mL^−1^ against *S. aureus* [59], respectively. Thus, the MIC result of EF implies the presence of strong antibacterial compounds in the extract fraction.

The level of antimicrobial susceptibility of natural products has been classified by some of the researchers based on the MIC value. According to Etame et al. [60] and Silva et al. [61], the antimicrobial activity of a particular extract is considered strong with MIC value lower than 0.100 mg mL^−1^, moderate with MIC value higher than 0.500 mg mL^−1^, and weak with MIC value higher than 1.00 mg mL^−1^. Moreover, the Clinical and Laboratory Standards Institute (CLSI) has suggested MIC breakpoint for commercial antibiotic, Chloramphenicol ≤ 8 µg mL^−1^ is considered susceptible against several bacteria such as *Staphylococcus* spp. in performing standard antimicrobial susceptibility test [62]. In the current work, the MIC values of *G. boninense* EF were lower than the guidelines reported in the previous works. Hence, extract of *G. boninense* from fruiting bodies affirmed strong antibacterial activity. The strong antibacterial activity in the current work also revealed the role of SPE fractionation in the pre-concentration of compounds of interest.

Interestingly, the highest inhibition zone was observed against CoNS, while it had a lower MIC value compared to MRSA. This phenomenon could be due to the SPE extract containing several antibacterial compounds with different molecular sizes and physico-chemical properties [63]. Small molecules diffuse better in agar medium, and non-polar compounds are hardly soluble in aqueous. Therefore, the particular compound responsible for inhibiting CoNS might be small in size but poorly soluble in an aqueous medium. The contradiction between agar diffusion and aqueous dilution test was reported previously [64,65,66].

### 2.3. Antibacterial Mode of Action of EF against MRSA

#### 2.3.1. The Effect of EF on Bacteria Morphology

**Figure 3:**
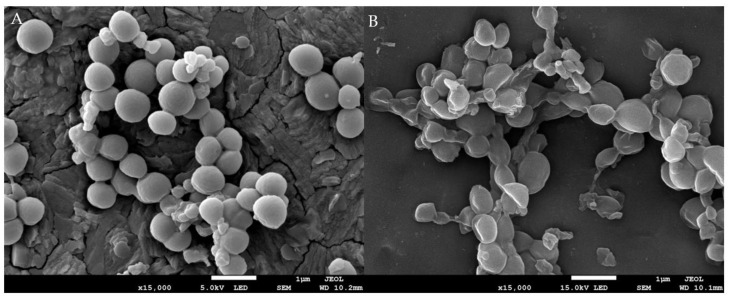
Images of the scanning electron microscope of MRSA before and after treatment with ethyl acetate *G. boninense* SPE elute fraction (EF): (**A**) control group of MRSA with 10% DMSO; (**B**) treatment group of MRSA with 1× MIC of EF. Both were incubated for 12 h at 37 °C.

The SEM images of MRSA after being treated with 1 × MIC and without treatment (control) are presented in Figure 3. The cellular morphology of untreated MRSA cells was regular round-shaped with a smooth surface and arranged in grape-like clusters (Figure 3A); however, EF-treated MRSA cells were pitted and shriveled with abnormality shape and collapsed surface (Figure 3B). EF has induced deformation and disruption of the cell membrane in MRSA. Morphology damage could lead to instability of cellular membrane and cell wall, which further causes leakage and losses of intracellular materials [67]. To our best knowledge, this is the first scanning electron microscope analysis reported on the effect of *Ganoderma* spp. extract on bacteria cell morphology. The similar morphological alteration was also reported on some basidiomycete fungi extract against different test microorganisms, such as extract of *Coriolus versicolor* against *S. aureus* and *S. enteritidis* [68], a semi-purified fraction of *Laetiporus sulphureus* extract against *E. coli* and *S. aureus* [69], and extract of *Lentinula edodes* against *S. mutans* and *P. aeruginosa* [70].

#### 2.3.2. The Effect of EF on Bacteria Cell Membrane Permeability

**Figure 4:**
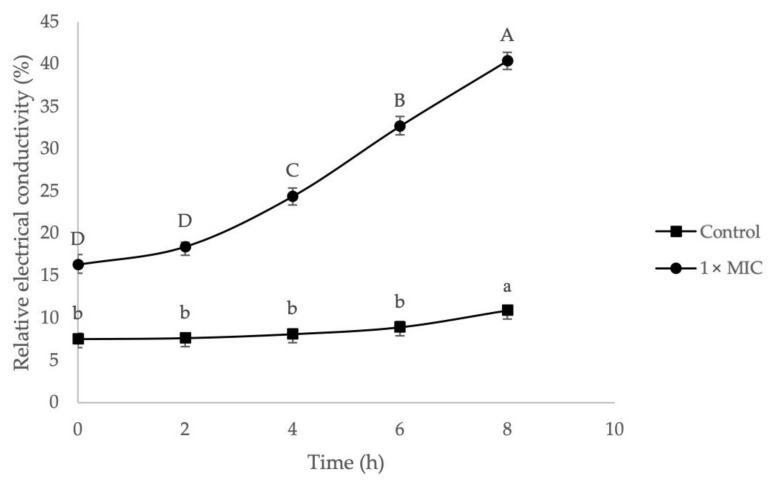
The effect of ethyl acetate *G. boninense* SPE elute fraction (EF) on the cell membrane permeability of MRSA. Different lower-case alphabets represent significantly different means of the control group at different time intervals (*p* < 0.05). Different upper-case alphabets represent significantly different means of the treatment group at different time intervals (*p* < 0.05).

The effect of 1× MIC of EF on bacterial cell membrane permeability was examined by measuring their relative electrical conductivity, and the result is illustrated in Figure 4. Results show the extract responded time-dependent effect as the relative electrical conductivity for MRSA was increased timely. The electrical conductivity was increased in the 8 h of treatment, ranging from 16.30 ± 1.21% to 40.39 ± 1.02%. In contrast, the control group only showed a slight increase from 7.51 ± 0.70% to 10.89 ± 0.20%. A significant increase of electrical conductivity (*p* < 0.05) in the control group can be observed only after 6 h of incubation. This might be due to normal lysis or natural death of bacterial cells. On the other hand, high relative electrical conductivity in the treatment group indicated leakage of electrolytes from the cell membrane and revealed the disruption in cell membrane permeability. The alteration of cell membrane permeability has interrupted the membrane’s barrier that allows passage of necessary ions and electrolytes for normal regulation of cell metabolism [71]. The mechanism of action in penetrating and altering the cell membrane of MRSA by furanone derivatives has been reported in detail [72].

#### 2.3.3. The Effect of EF on Bacteria Cell Membrane Integrity

**Figure 5:**
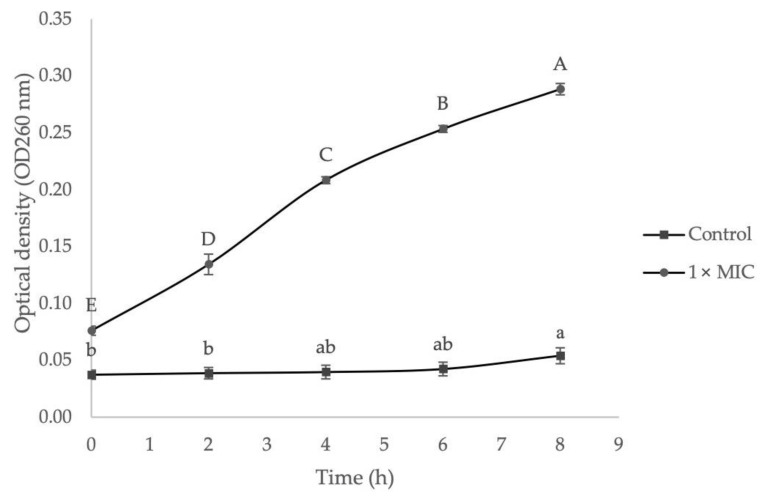
The effect of ethyl acetate *G. boninense* SPE elute fraction (EF) on the cell membrane integrity of MRSA. Different lower-case alphabets represent significantly different means of the control group at different time intervals (*p* < 0.05). Different upper-case alphabets represent significantly different means of the treatment group at different time intervals (*p* < 0.05).

The effect of EF on bacteria cell membrane integrity was investigated by measuring the absorbance value of 260 nm absorbing materials, which were released from the intracellular membrane after bacteria cells were exposed to 1× MIC of extract. As presented in Figure 5, the absorbance values at 260 nm treated with 1× MIC of extract were increased significantly for MRSA, ranging from 0.076 ± 0.004 nm to 0.288 ± 0.005 nm after 8 h of treatment. In contrast, the 260 nm absorbance values of the control group showed only a slight increase, ranging from 0.037 ± 0.004 nm to 0.054 ± 0.007 nm, which significant increase (*p* < 0.05) was only observed after 4 h and 6 h. This situation could be due to the normal lysis and cells death over time. In this study, the cell membrane integrity was expressed by recording the absorbance value of 260 nm since genetic materials such as nucleic acids, RNA, or DNA have a maximum UV absorption peak at 260 nm. A higher 260 nm absorbance value in supernatant indicates higher nucleic acid content was released from cells. Based on the result, MRSA showed a significant increase of 260 nm absorbance value after being treated with 1× MIC of *G. boninense* SPE extract and confirmed the release of intracellular genetic materials that resided in the interior membranes to the outer solution. The leakage of these important materials reveals irreversible damage to the membrane integrity and causes cellular malfunction and further cell death [73].

## 3. Materials and Methods

### 3.1. Preparation of G. boninense Crude Extract and Clean-Up

Wild fruiting bodies of *G. boninense* were collected from an oil palm estate in Sabah, Malaysia, identified using molecular technique, and extracted using absolute ethyl acetate (Fisher Chemical, Pittsburgh, PA, USA) as described previously by Chan and Chong [17]. The crude extract obtained was prepared in 20 mg/mL, and the normal phase Strata^®^ Si-1 (55 µm, 70 Å) from Phenomenex^®^ (Torrance, CA, USA) was used for crude extract clean-up. The SPE cartridge was preconditioned with 1 mL of ethyl acetate to activate the sorbent bed by solvation. Then, 600 µL of the extract was loaded slowly into the cartridge and allowed the sample to associate with the sorbent before eluting. The unretained fractions were collected and labeled as “Flush Fraction”. After 5 min, the cartridge was washed three times with 1.2 mL of methanol to elute the retained compounds. The retained fractions were collected and labeled as “Elute Fraction”. Both fractions were dried under a gentle stream of nitrogen gas. The dried fractions were kept at −20 °C until further use.

### 3.2. Chromatographic Separation and Mass Spectra Detection of G. boninense SPE Extract

Separation of the SPE extract (EF) was performed using an HPLC system (Dionex UltiMate 3000, Thermo Fisher Scientific, Waltham, MA, USA) coupled with ion trap mass spectrometry (Bruker Amazon SL, USA). The chromatography and spectrometry conditions were set as described by Wu et al. [74] with some modification. Hypersil TM ODS C18 column (4.6 mm × 30 mm, 3 μm; Thermo Fisher Scientific, Waltham, MA, USA) was used, and the mobile phase consisted of HPLC grade acetonitrile (Fisher Chemical, Pittsburgh, PA, USA) (A) and 0.2% formic acid in HPLC grade water (Fisher Chemical, Pittsburgh, PA, USA) (B) modified with gradient elution of 0 min (20% A), 2 min (30% A), 5 min (65% A), 20 min (75% A), 28 min (90% A), 48 min (95% A), and 50 min (95% A). Metabolites separated in the LC system were further detected using ion trap mass spectrometry.

### 3.3. Evaluation of Antibacterial Activity of G. boninense SPE Extract

#### 3.3.1. Antibacterial Disc Diffusion Assay

Clinical isolate bacterial samples, *S. pyogenes*, CoNS, and MRSA were obtained from the Genetic laboratory of Universiti Malaysia Sabah (UMS) and sub-cultured for disc diffusion assay to evaluate the antibacterial activity of SPE extract of *G. boninense* as described by CLSI guidelines [75]. In brief, 100 μL of freshly grown bacterial suspensions containing 1–2 × 10^8^ CFU/mL was spread evenly on Muller-Hinton agar (MHA) plates using a sterile cotton swab. Prior to placing the extracted disc, the SPE extracts were re-dissolved in 10% Dimethyl Sulfoxide (DMSO) (Fisher Chemical, Pittsburgh, PA, USA) by dilution using sterile distilled water. Sterile disc of Whatman^®^ No.3 filter paper (6 mm diameter) which loaded with SPE extract (20 mg/disc), was further placed on top of the MHA plates. Two control discs that contained Chloramphenicol (30 μg/disc) and 10% DMSO were used as a positive and negative control. The MHA plates were incubated at 37 °C for 24 h. The diameter of the zone of inhibition (mm) was measured to determine the antibacterial activity of each fraction.

#### 3.3.2. Determination of Minimum Inhibition Concentration (MIC)

The microdilution method as described by Paula et al. [76] with slight modification was adopted in the current work to determine the minimum inhibitory concentration (MIC) of the SPE extract, which has shown good antibacterial activity. The 96 well microtiter plate was filled with 100 μL of nutrient broth (NB), followed by 100 μL of extract (40 mg mL^−1^). The solution in well was diluted in a series of two-fold dilutions and resulted in concentrations ranging from 0.625 to 20 mg mL^−1^ in the subsequent wells in the same row. The final concentration of suspension in each well was adjusted to 5 × 10^5^ CFU/mL with 50 μL of bacterial suspension. The microtiter plate was included with a set of three controls: Chloramphenicol (30 μg) as positive control; 10% DMSO as negative control; and all solutions except the extract to ensure bacteria growth. The plate was incubated at 37 °C for 24 h before being read at 590 nm using a microplate reader (Multiskan™GO, Thermo Fisher Scientific, Waltham, MA, USA).

### 3.4. Mode of Action of G. boninense SPE Extract against Selected Pathogen

#### 3.4.1. Observation of Morphological Changes under Scanning Electron Microscope (SEM)

The sample preparation for SEM (EVO MA 10, Carl Zeiss, Germany) was conducted according to Zhang et al. [73] with slight modification. Freshly cultured bacterial suspension of MRSA was adjusted to approximately 1–2 × 10^8^ CFU/mL. The bacterial cells were centrifuged for 5 min at 2500× *g* and further treated with 1 × MIC of SPE extract (EF). Meanwhile, bacteria treated with 10% DMSO without extract were used as control. The sample was incubated for 12 h at 37 °C and then washed twice with 0.1 M PBS (pH 7.4) by centrifugation for 10 min at 5000× *g* followed by fixation in 2.5% (*v*/*v*) glutaraldehyde for 2 h at 4 °C. The fixed samples were dehydrated in serial of ethanol gradients (30%, 50%, 80%, 90%, and 100%) for 10 min each and lastly suspended in tertiary butyl alcohol. The specimens were dried by freeze dryer (Scanvac Labogene^®^ model: Cool Safe 110-4) and sputter-coated for SEM observation.

#### 3.4.2. Measurement of Cell Membrane Permeability

The effect of treatment on bacterial cell membrane permeability was expressed in terms of relative electrical conductivity as described by Patra and Baek [77] with minor modification. Freshly cultured bacterial suspension of MRSA was spectrophotometrically adjusted to approximately 1–2 × 10^8^ CFU/mL and centrifuged at 2800× *g* for 10 min. Bacterial cells were washed with 5% glucose solution (*w*/*v*) until their electrical conductivity was close to that 5% glucose solution, which is an indication of isotonic bacteria. Electric conductivity was measured using an electrical conductivity meter (EC300A, EcoSense, Yellow Springs, OH, USA). Electrical conductivity of SPE extract (1× MIC EF) with 5% glucose was marked as L_1_. Isotonic bacteria suspension added with SPE extract (1× MIC EF) was mixed and incubated at 37 °C for 8 h. Their electrical conductivity was measured at 2 h of intervals and marked as L_2_. Electrical conductivity of isotonic bacteria in 5% glucose solution was killed using boiling water, used as control, and marked as L_0_. The permeability of the cell membrane was calculated as follows:

(1) $$\mathrm{Relative}\;\mathrm{electrical}\;\mathrm{conductivity}\;(\%)=\frac{\left(L2-L1\right)}{L0}\times 100$$

#### 3.4.3. Measurement of Cell Membrane Integrity

The effect of SPE fraction (EF) on bacterial cell membrane integrity was determined by measuring the release of 260 nm absorbing materials from test microorganisms in accordance with Patra and Baek [77] with slight modification. Freshly grown bacterial suspension was spectrophotometrically adjusted to 1–2 × 10^8^ CFU/mL and harvested by centrifugation at 5000× *g* for 15 min followed by three times wash with 0.1 M PBS (pH 7.4). The bacterial cells were resuspended in PBS solution and added with 1× MIC of SPE extract (EF) followed by incubation at 37 °C for 8 h. The bacterial suspension was added with 10% DMSO but without SPE extract and served as control. Bacterial samples were collected and centrifuged at 3500× *g* every 2 h intervals. The supernatant was measured at 260 nm using a spectrophotometer (Multiskan™GO, Thermo Fisher Scientific, Waltham, MA, USA). MIC of extract in PBS without bacterial cells served as blank for the treatment group while the untreated group was blanked with PBS.

### 3.5. Statistical Analysis

All experiments were conducted in triplicate, and the results are expressed as mean ± standard deviation (SD). One-way analysis of variance (ANOVA) and post hoc tests by Tukey’s test was performed using SPSS version 27 (IBM Corp, Armonk, NY, USA). Value of *p* < 0.05 was recorded as a statistically significant difference between means.

## 4. Conclusions

The significant inhibitory effect of the ethyl acetate *G. boninense* SPE elute fraction (EF) against the selected Gram-positive bacteria evidenced SPE method has successfully preconcentrated the antibacterial compounds from the *G. boninense* crude extract. The presence of metabolites in the extract was identified, and the compounds responsible for the antibacterial activity were also reported by referring to established literature. Furthermore, results obtained from this fraction in disc diffusion and broth microdilution assays have affirmed strong antibacterial performance against those bacteria. Investigation on the mode of action of EF against MRSA cells gave an insight that this fraction induces irreversible membrane damage effects associated with disruption of cell membrane permeability and integrity by causing leakage of intracellular electrolytes and 260 nm absorbing materials. Moreover, the membrane damage was also supported by the SEM observation, where morphological alteration was observed in the physical appearance of the tested bacterial cells after being exposed to the extract. The change of membrane is lethally corresponding to the cellular lysis and death. Therefore, the antibacterial potency of a semi-purified fraction of *G. boninense* extract should be highlighted for the potential development of new therapeutic agents and diversity exploration of other biological potentials in the future.

## Figures and Tables

**Table 1:** Identified compounds and metabolites profile of ethyl acetate *G. boninense* SPE elute fraction (EF) by non-targeted LC–Ion Trap–MS in negative ionization mode.

Peak No	RT (Min)	Measured *m/z*	Putative Identity	Molecular Formula	Metabolite Class	Peak Height	References
1	0.81	656.7744	N-(3-acetamido-5-carbamoyl-2,4,6-triiodobenzoyl)glycine	C_12_H_10_I_3_N_3_O_5_	Amino acid amide	1.5 × 10^7^	NA
2	7.11	515.1974	Pyroglu-gln-gly-ser-asn	C_19_H_29_N_7_O_10_	Peptide	8.46 × 10^7^	NA
3	7.54	499.1844	Idarubicinol	C_26_H_29_NO_9_	Glycoside	6.63 × 10^7^	NA
4	8.26	585.2618	Carbanilic acid, diester with 2,2′-((2-butoxy-3-methoxyphenethyl)nitrilo)diethanol, hydrochloride	C_31_H_40_CIN_3_O_6_	Heterocyclic compound	7.07 × 10^7^	NA
5	9.33	499.1782	5-*O*-(Indol-3-ylacetyl-myo-inositol) d-galactoside	C_22_H_29_NO_12_	Glycoside	4.53 × 10^7^	NA
6	10.65	341.0627	Aristolochic acid	C_17_H_11_NO_7_	Isoquinoline alkaloid	3.70 × 10^7^	[32]
7	11.53	543.2351	Ataralgin	C_26_H_33_N_5_O_8_	Amine	3.55 ×10^7^	NA
8	12.50	483.1897	But-2-enedioic acid; N-ethyl-N-[[4-fluoro-2-(4-fluorophenyl)phenyl]methoxy]-2-phenyl-ethanamine	C_27_H_27_F_2_NO_5_	Benzhydryl compound	2.96 × 10^7^	NA
9	13.56	295.0576	Aminoimidazole ribotide	C_8_H_14_N_3_O_7_P	Glycoside	1.81 × 10^7^	[33]
10	16.66	517.2613	Lysine Sulfonamide 11v	C_27_H_39_N_3_O_5_S	Peptide hydrolases	5.40 × 10^7^	[34,35]
11	17.15	517.2434	Deglucohyrcanoside	C_28_H_38_O_9_	Glycoside	3.72 × 10^7^	NA
12	22.64	469.2465	Carbocyclic puromycin	C_23_H_31_N_7_O_4_	Glycoside	1.56 × 10^7^	[36]
13	27.85	279.0042	Arylmercury	C_6_H_5_Hg	Organomercury compound	1.44 × 10^8^	NA
14	30.48	299.0668	Fenbendazole	C_15_H_13_N_3_O_2_S	Heterocyclic compound	1.99 × 10^8^	[37,38]
15	30.93	281.05078	4,5-Methylenedioxy-6-hydroxyaurone	C_16_H_10_O_5_	Flavonoid	1.28 × 10^8^	NA
16	32.86	313.1277	Acetylcaranine	C_18_H_19_NO_4_	Alkaloids	1.37 × 10^7^	[39]
17	33.26	585.2877	Tigecycline	C_29_H_39_N_5_O_8_	Polycyclic aromatic hydrocarbons	1.62 × 10^7^	[40]
18	35.52	383.0788	Tetraphenylarsonium	C_24_H_20_As	Arsenicals	1.56 × 10^7^	NA
19	35.87	371.2174	Tamoxifen	C_26_H_29_NO	Benzylidene compounds	7.5 × 10^6^	[38,41]
20	39.06	385.1992	O-Methylandrocymbine	C_22_H_27_NO_5_	Alkaloids	1.86 × 10^7^	NA
21	42.49	355.21083	Spirasine I	C_22_H_29_NO_3_	Terpenoid alkaloids	2.35 × 10^7^	NA
22	42.92	399.2483	Spiramine A	C_24_H_33_NO_4_	Terpenoid alkaloids	2.11 × 10^7^	NA
23	46.75	369.2216	Cilostazol	C_20_H_27_N_5_O_2_	Quinolinone	3.0 × 10^7^	NA

References show compounds with known antimicrobial activity as reported in the literature. NA—no literature was found reporting on its antimicrobial activity.

**Table 2:** Inhibition zones of SPE flush and elute fractions of ethyl acetate extracts of *G. boninense* fruiting bodies against tested clinical bacterial isolates.

Bacterial Strains	Inhibition Zones (mm)		
	Flush Fraction (FF)	Elute Fraction (EF)	Chloramphenicol
*S. pyogenes*	8.75 ± 0.04 ^c^	30.75 ± 0.03 ^a^	22.58 ± 0.05 ^b^
CoNS	20.42 ± 0.04 ^c^	47.25 ± 0.07 ^a^	30.33 ± 0.03 ^b^
MRSA	14.83 ± 0.01 ^c^	41.08 ± 0.04 ^a^	26.67 ± 0.03 ^b^

Each disc of the test consists of 20 mg of extract. Chloramphenicol (30 μg/disc) was used as a positive control in this bioassay and 10% DMSO as negative control. No inhibition was observed on the negative control disc. Data are expressed as mean ± SD, *n* = 3. Different alphabets within rows represent statistically significant differences at *p* < 0.05 by one-way ANOVA and Tukey’s test.

**Table 3:** Minimum inhibitory concentration (MIC) of the SPE elute fraction of ethyl acetate *G. boninense* fruiting bodies extract (EF) and Chloramphenicol against tested clinical bacterial isolates.

Bacterial Strains	MIC	
	EF (mg mL^−1^)	Chloramphenicol (µg mL^−1^)
*S. pyogenes*	0.156	7.50
CoNS	0.156	3.75
MRSA	0.078	3.75

## Data Availability

The data in this manuscript will be made available to public.

## Sample Availability

Interested parties shall contact the corresponding author.

## References

[1] Wang L., Li J.Q., Zhang J., Li Z.M., Liu H.G., Wang Y.Z. (2020). Traditional uses, chemical components and pharmacological activities of the genus *Ganoderma* P. Karst.: A review. RSC Adv..

[2] Miao W.G., Tang C., Ye Y., Quinn R.J., Feng Y. (2019). Traditional Chinese medicine extraction method by ethanol delivers drug-like molecules. Chin. J. Nat. Med..

[3] Wachtel-Galor S., Yuen J., Buswell J.A., Benzie I.F.F., Watchtel-Galor S. (2011). Ganoderma lucidum (Lingzhi or Reishi): A medicinal mushroom. Herbal Medicine: Biomolecular and Clinical Aspects.

[4] Huang W.C., Chang M.S., Huang S.Y., Tsai C.J., Kuo P.H., Chang H.W., Kao M.C. (2019). Chinese herbal medicine *Ganoderma tsugae* displays potential anti-cancer efficacy on metastatic prostate cancer cells. Int. J. Mol. Sci..

[5] Yang Y., Zhang H., Zuo J., Gong X., Yi F., Zhu W., Li L. (2019). Advances in research on the active constituents and physiological effects of *Ganoderma lucidum*. Biomed. Dermatol..

[6] Li L.F., Liu H.B., Zhang Q.W., Li Z.P., Wong T.L., Fung H.Y., Han Q.B. (2018). Comprehensive comparison of polysaccharides from *Ganoderma lucidum* and *G. sinense*: Chemical, antitumor, immunomodulating and gut-microbiota modulatory properties. Sci. Rep..

[7] Sharma C., Bhardwaj N., Sharma A., Tuli H.S., Batra P., Beniwal V., Sharma A.K. (2019). Bioactive metabolites of *Ganoderma lucidum*: Factors, mechanism and broad spectrum therapeutic potential. J. Herb. Med..

[8] Lin A.X., Chan G., Hu Y., Ouyang D., Ung C.O.L., Shi L., Hu H. (2018). Internationalization of traditional Chinese medicine: Current international market, internationalization challenges and prospective suggestions. Chin. Med..

[9] Li S., Dong C., Wen H.A., Liu X. (2016). Development of Ling-zhi industry in China emanated from the artificial cultivation in the Institute of Microbiology, Chinese Academy of Sciences (IMCAS). Mycology.

[10] Heleno S.A., Barros L., Martins A., Queiroz M.J.R., Santos-Buelga C., Ferreira I.C. (2012). Fruiting body, spores and in vitro produced mycelium of *Ganoderma lucidum* from Northeast Portugal: A comparative study of the antioxidant potential of phenolic and polysaccharide extracts. Food Res. Int..

[11] Zhou X.W., Su K.Q., Zhang Y.M. (2012). Applied modern biotechnology for cultivation of *Ganoderma* and development of their products. Appl. Microbiol. Biotechnol..

[12] Moradali M.F., Mostafavi H., Hejaroude G.A., Tehrani A.S., Abbasi M., Ghods S. (2006). Investigation of potential antibacterial properties of methanol extracts from fungus *Ganoderma applanatum*. Chemotherapy.

[13] Smania E.D.F.A., Delle Monache F., Yunes R.A., Paulert R., Smania A. (2007). Antimicrobial activity of methyl australate from *Ganoderma australe*. Rev. Bras. Farmacogn..

[14] Osinska-Jaroszuk M., Jaszek M., Mizerska-Dudka M., Blachowicz A., Rejczak T.P., Janusz G., Kandefer-Szerszen M. (2014). Exopolysaccharide from *Ganoderma applanatum* as a promising bioactive compound with cytostatic and antibacterial properties. Biomed. Res. Int..

[15] Ma K., Li L., Bao L., He L., Sun C., Zhou B., Liu H. (2015). Six new 3, 4-seco-27-norlanostane triterpenes from the medicinal mushroom *Ganoderma boninense* and their antiplasmodial activity and agonistic activity to LXRβ. Tetrahedron.

[16] Ismail K., Abdullah S., Chong K.P. (2014). Screening for potential antimicrobial compound from *Ganoderma boninense* against selected foodborne and skin disease pathogens. Int. J. Pharm. Pharm. Sci..

[17] Chan Y.S., Chong K.P. (2020). Antimicrobial activity and metabolite analysis of *Ganoderma boninense* fruiting body. J. Pure Appl. Microbiol..

[18] Chan Y.S., Chong K.P. (2020). Phytochemical investigation and antioxidant activity of *Ganoderma boninense*. Malays. J. Biochem. Mol. Biol..

[19] Abdullah S., Oh Y.S., Kwak M.K., Chong K.P. (2021). Biophysical characterization of antibacterial compounds derived from pathogenic fungi *Ganoderma boninense*. J. Microbiol..

[20] Abdullah S., Jang S.E., Kwak M.K., Chong K.P. (2020). *Ganoderma boninense* mycelia for phytochemicals and secondary metabolites with antibacterial activity. J. Microbiol..

[21] Ramzi A.B., Me M.L.C., Ruslan U.S., Baharum S.N., Muhammad N.A.N. (2019). Insight into plant cell wall degradation and pathogenesis of *Ganoderma boninense* via comparative genome analysis. PeerJ.

[22] Govender N.T., Mahmood M., Seman I.A., Wong M.Y. (2017). The phenylpropanoid pathway and lignin in defense against *Ganoderma boninense* colonized root tissues in oil palm (*Elaeis guineensis* Jacq.). Front. Plant Sci..

[23] Muniroh M.S., Nusaibah S.A., Vadamalai G., Siddique Y. (2019). Proficiency of biocontrol agents as plant growth promoters and hydrolytic enzyme producers in *Ganoderma boninense* infected oil palm seedling. Curr. Plant Biol..

[24] Nusaibah S.A., Musa H., Shah M.M., Sharif U., Buhari T.R. (2019). A review report on the mechanism of *Trichoderma* spp. as biological control agent of basal stem root (BSR) disease of *Elaeis guineensis*. Trichoderma—The Most Widely Used Fungicide.

[25] Alexander A., Sipaut C.S., Dayou J., Chong K.P. (2017). Oil palm roots colonization by *Ganoderma boninense*: An insight study using scanning electron microscopy. J. Oil Palm Res..

[26] Centers for Disease Control and Prevention (CDC) (2019). Antibiotic Resistance Threats in United States.

[27] World Health Organization (WHO). https://www.euro.who.int/en/health-topics/disease-prevention/antimicrobial-resistance/about-amr/global-trends-bacteria.

[28] Kali A. (2015). Antibiotics and bioactive natural products in treatment of methicillin resistant *Staphylococcus aureus*: A brief review. Pharmacogn. Rev..

[29] Azwanida N.N. (2015). A review on the extraction methods use in medicinal plants, principle, strength and limitation. Med. Aromat. Plants.

[30] Faraji M., Yamini Y., Gholami M. (2019). Recent advances and trends in applications of solid-phase extraction techniques in food and environmental analysis. Chromatographia.

[31] Otles S., Kartal C. (2016). Solid-phase extraction (SPE): Principles and applications in food samples. Acta Sci. Pol. Technol. Aliment..

[32] Bartha G.S., Toth G., Horvath P., Kiss E., Papp N., Kerenyi M. (2019). Analysis of aristolochlic acids and evaluation of antibacterial activity of *Aristolochia clematitis* L.. Biol. Futur..

[33] Kim A., Wolf N.M., Zhu T., Johnson M.E., Deng J., Cook J.L., Fung L.W.M. (2015). Identification of *Bacillus anthracis* PurE inhibitors with antimicrobial activity. Bioorg. Med. Chem..

[34] Qadir M.A., Ahmed M., Khaleeq A. (2016). Synthesis, antibacterial and antifungal possession of amino acids containing sulfonamide moieties. Pak. J. Pharm. Sci..

[35] Stranix B.R., Lavallée J.F., Sévigny G., Yelle J., Perron V., LeBerre N., Wu J.J. (2006). Lysine sulfonamides as novel HIV-protease inhibitors: Nε-acyl aromatic α-amino acids. Bioorg. Med. Chem..

[36] Vince R., Daluge S., Brownell J. (1986). Carbocyclic purmocyin: Synthesis and inhibition of protein biosynthesis. J. Med. Chem..

[37] de Oliveira H.C., Joffe L.S., Simon K.S., Castelli R.F., Reis F.C., Bryan A.M., Rodrigues M.L. (2020). Fenbendazole controls in vitro growth, virulence potential, and animal infection in the *Cryptococcus* model. Antimicrob. Agents Chemother..

[38] Miro-Canturri A., Ayerbe-Algaba R., Smani Y. (2019). Drug repurposing for the treatment of bacterial and fungal infections. Front. Microbial..

[39] Ločárek M., Nováková J., Klouček P., Hošt’álková A., Kokoška L., Gábrlová L., Cahlíková L. (2015). Antifungal and antibacterial activity of extracts and alkaloids of selected *Amaryllidaceae* species. Nat. Prod. Commun..

[40] Livermore D.M. (2005). Tigecycline: What is it, and where should it be used?. J. Antimicrob. Chemother..

[41] Flores R., Insel P.A., Nizet V., Corriden R. (2016). Enhancement of neutrophil antimicrobial activity by the breast cancer drug tamoxifen. FASEB J..

[42] Paulsen J.E., Sundby-Hall K., Endresen L., Rugstad H.E., Reichelt K.L., Elgjo K. (1991). The peptide pyroGlu-Gln-Gly-Ser-Asn, isolated from mouse liver, inhibits growth of rat hepatoma cells in vitro. Carcinogenesis.

[43] Fukushima T., Kawai Y., Urasaki Y., Yoshida A., Ueda T., Nakamura T. (1994). Influence of idarubicinol on the antileukemic effect of idarubicin. Leuk. Res..

[44] Rimpelová S., Zimmermann T., Drašar P.B., Dolenský B., Bejček J., Kmoníčková E., Jurášek M. (2021). Steroid glycosides hyrcanoside and deglucohyrcanoside: On Isolation, structural identification, and anticancer activity. Foods.

[45] Li L., Shen Y.M., Yang X.S., Zuo G.Y., Shen Z.Q., Chen Z.H., Hao X.J. (2002). Antiplatelet aggregation activity of diterpene alkaloids from *Spiraea japonica*. Eur. J. Pharmacol..

[46] Nakashima H., Watanabe K., Umegaki H., Suzuki Y., Kuzuya M. (2018). Cilostazol for the prevention of pneumonia: A systematic review. Pneumonia.

[47] Zhou B., Xiao J.F., Tuli L., Ressom H.W. (2012). LC-MS based metabolomics. Mol. Biosyst..

[48] (2022). The European Committee on Antimicrobial Susceptibility Testing (EUCAST). https://eucast.org/clinical_breakpoints/.

[49] Chan Y.S. (2021). Antibacterial, Antioxidant and Phytochemical Analysis of *Ganoderma boninense*. Master’s Thesis.

[50] Breijyeh Z., Jubeh B., Karaman R. (2020). Resistance of gram-negative bacteria to current antibacterial agents and approaches to resolve it. Molecules.

[51] Blair J.M., Richmond G.E., Piddock L.J. (2014). Multidrug efflux pumps in gram-negative bacteria and their role in antibiotic resistance. Futur. Microbiol..

[52] El-Fayoumy E.A., Shanab S.M., Gaballa H.S., Tantawy M.A., Shalaby E.A. (2021). Evaluation of antioxidant and anticancer activity of crude extract and different fractions of *Chlorella vulgaris* axenic culture grown under various concentrations of copper ions. BMC Complement. Med. Ther..

[53] Dugheri S., Marrubini G., Mucci N., Cappelli G., Bonari A., Pompilio I., Arcangeli G. (2020). A review of micro-solid-phase extraction techniques and devices applied in sample pretreatment coupled with chromatographic analysis. Acta Chromatogr..

[54] Andrade-Eiroa A., Canle M., Leroy-Cancellieri V., Cerda V. (2016). Solid-phase extraction of organic compounds: A critical review part ii. TrAC Trend Anal. Chem..

[55] Zhou S., Zhang J., Ma F., Tang C., Tang Q., Zhang X. (2018). Investigation of lignocellulolytic enzymes during different growth phases of *Ganoderma lucidum* strain using genomic, transcriptomic and secretomic analyses. PLoS ONE.

[56] Wubshet S.G., Johansen K.T., Nyberg N.T., Jaroszewski L.W. (2012). Direct 13C NMR detection in HPLC hyphenation mode: Analysis of *Ganoderma lucidum* terpenoids. J. Nat. Prod..

[57] Quereshi S., Pandey A.K., Sandhu S.S. (2010). Evaluation of antibacterial activity of different *Ganoderma lucidum* extracts. J. Sci. Res..

[58] Yoon S.Y., Eo S.K., Kim Y.S., Lee C.K., Han S.S. (1994). Antimicrobial activity of *Ganoderma lucidum* extract alone and in combination with some antibiotics. Arch. Pharm. Res..

[59] Mothana R.A., Jansen R., Jülich W.D., Lindequist U. (2000). Ganomycins A and B, new antimicrobial farnesyl hydroquinones from the basidiomycete *Ganoderma pfeifferi*. J. Nat. Prod..

[60] Etame R.E., Mouokeu R.S., Pouaha C.L.C., Kenfack I.V., Tchientcheu R., Assam J.P.A., Ngane R.A.N. (2018). Effect of fractioning on antibacterial activity of *Enantia chlorantha* Oliver (Annonaceae) methanol extract and mode of action. Evid. Based Complement. Alternat. Med..

[61] Silva A.C.O., Santana E.F., Saraiva A.M., Coutinho F.N., Castro R.H.A., Pisciottano M.N.C., Albuquerque U.P. (2013). Which approach is more effective in the selection of plants with antimicrobial activity?. Evid. Based Complement. Alternat. Med..

[62] Clinical and Laboratory Standards Institute (CLSI) (2020). Performance Standards for Antimicrobial Susceptibility Testing. https://www.nih.org.pk/wp-content/uploads/2021/02/CLSI-2020.pdf.

[63] Eloff J.N. (2019). Avoiding pitfalls in determining antimicrobial activity of plant extracts and publishing the results. BMC Complement. Altern. Med..

[64] Donaldson J.R., Warner S.L., Cates R.G., Gary Y.D. (2005). Assessment of antimicrobial activity of fourteen essential oils when using dilution and diffusion methods. Pharm. Biol..

[65] Burman S., Bhattacharya K., Mukherjee D., Chandra G. (2018). Antibacterial efficacy of leaf extracts of *Combretum album* Pers. against some pathogenic bacteria. BMC Complement. Altern. Med..

[66] Kim G., Gan R.Y., Zhang D., Farha A.K., Habimana O., Mayumengwana V., Corke H. (2020). Large scale screening of 239 traditional Chinese medicinal plant extracts for their antibacterial activities against multidrug resistant *Staphylococcus aureus* and cytotoxic activities. Pathogens.

[67] Bajpai V.K., Sharma A., Baek K.H. (2013). Antibacterial mode of action of *Cudrania tricuspidata* fruit essential oil, affecting membrane permeability and surface characteristics of food-borne pathogens. Food Control.

[68] Matijašević D., Pantić M., Rašković B., Pavlović V., Duvnjak D., Sknepnek A., Nikšić M. (2016). The antibacterial activity of *Coriolus versicolor* methanol extract and its effect on ultrastructural changes of *Staphylococcus aureus* and *Salmonella enteritidis*. Front. Microbial..

[69] Younis A.M., Yosri M., Stewart J.K. (2019). In vitro evaluation of pleiotropic properties of wild mushroom *Laetiporus sulphureus*. Ann. Agric. Sci..

[70] Shang X., Muthu M., Keum Y.S., Chun S., Gopal J. (2016). An agile, simplified and sonication mediated one-pot aqueous extraction and antibacterial assessment of predominant Korean mushrooms. RSC Adv..

[71] Sadiq M.B., Tarning J., Aye Cho T.Z., Anal A.K. (2017). Antibacterial activities and possible modes of action of *Acacia nilotica* (L.) Del. against multidrug-resistant Escherichia coli and Salmonella. Molecules.

[72] Sharafutdinoy I.S., Pavlova A.S., Akhatova F.S., Khabibrakhmanova A.M., Rozhina E.V., Romanva Y.J., Kayumov A.R. (2019). Unraveling the molecular mechanism of selective antimicrobial activity of 2(5H)-furanone derivatives against *Staphylococcus aureus*. Int. J. Mol. Sci..

[73] Zhang J., Ye K.P., Zhang X., Pan D.D., Sun Y.Y., Cao J.X. (2017). Antibacterial activity and mechanism of action of black pepper essential oil on meat-borne *Escherichia coli*. Front. Microbial..

[74] Wu L., Liang W., Chen W., Li S., Cui Y., Qi Q., Zhang L. (2017). Screening and analysis of the marker components in *Ganoderma lucidum* by HPLC and HPLC-MS^n^ with the aid of chemometrics. Molecules.

[75] CLSI (2011). Performance Standards for Antimicrobial Disk Susceptibility Testing: Twenty-First Informational Supplement.

[76] Paula C.C., Martins D.T.O., Arunachalam K., Balogun S.O., Borges Q.I., Picone M.G., Barros W.M., Prado R.M.S. (2018). Anti-microbial screening of medicinal plants popularly used Mato Grosso for treating infections: Advances on the evaluation of *Conyza bonariensis* (L.) Cronquist in vitro and in vivo antibacterial activity. Pharmacogn. J..

[77] Patra J.K., Baek K.H. (2016). Antibacterial activity and action mechanism of the essential oil from *Enteromorpha linza* L. against foodborne pathogenic bacteria. Molecules.

